# A Comprehensive Review on the Synthesis, Characterization, and Biomedical Application of Platinum Nanoparticles

**DOI:** 10.3390/nano9121719

**Published:** 2019-12-02

**Authors:** Muniyandi Jeyaraj, Sangiliyandi Gurunathan, Muhammad Qasim, Min-Hee Kang, Jin-Hoi Kim

**Affiliations:** Department of Stem Cell and Regenerative Biotechnology and Humanized Pig Center (SRC), Konkuk Institute of Technology, Konkuk University, Seoul 05029, Korea; muniyandij@yahoo.com (M.J.); gsangiliyandi@yahoo.com (S.G.); qasimattock@gmail.com (M.Q.); pocachippo@gmail.com (M.-H.K.)

**Keywords:** synthesis, characterization, biomedical applications, cytotoxicity, anticancer, combination therapy

## Abstract

Platinum nanoparticles (PtNPs) are noteworthy scientific tools that are being explored in various biotechnological, nanomedicinal, and pharmacological fields. They are unique because of their large surface area and their numerous catalytic applications such as their use in automotive catalytic converters and as petrochemical cracking catalysts. PtNPs have been widely utilized not only in the industry, but also in medicine and diagnostics. PtNPs are extensively studied because of their antimicrobial, antioxidant, and anticancer properties. So far, only one review has been dedicated to the application of PtNPs to nanomedicine. However, no studies describe the synthesis, characterization, and biomedical application of PtNPs. Therefore, the aim of this review is to provide a comprehensive assessment of the current knowledge regarding the synthesis, including physical, chemical, and biological and toxicological effects of PtNPs on human health, in terms of both in vivo and in vitro experimental analysis. Special attention has been focused on the biological synthesis of PtNPs using various templates as reducing and stabilizing agents. Finally, we discuss the biomedical and other applications of PtNPs.

## 1. Introduction

**Figure 1:**
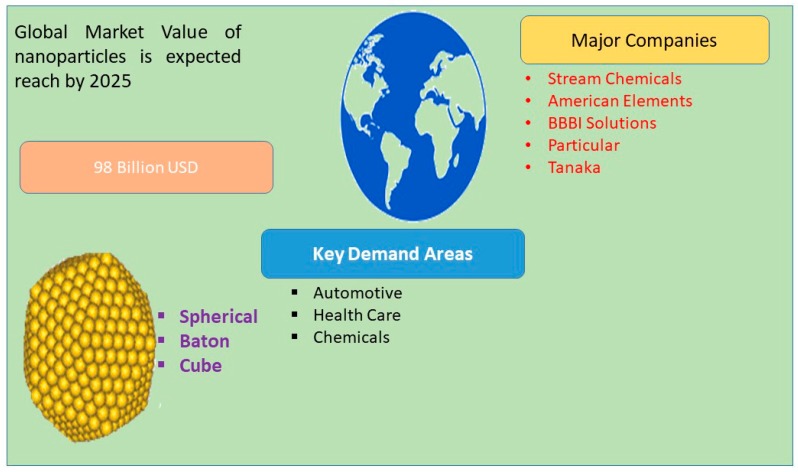
Global market value of nanoparticles in 2025.

Nanotechnology is a burgeoning field and is widely applied to biomedical engineering and nanomedicine. Numerous technologies are involved in the fabrication of nanomaterials from various sources such as physical, chemical, and biological materials, and different strategies are used to maximize the production of nanomaterials such as the use of different raw materials, temperature, and pH. The global market has a high demand for nanoparticles (NPs), and it is expected that this demand will reach 98 billion dollars by 2025 (Figure 1).

NPs are complex molecules composed of triple-layered surfaces: the surface layer, the shell, and the core [1]. Among other metallic NPs, noble nanomaterials have been used extensively for the applications in fuel cells, organic catalysis, water gas shift reactions, selective oxidation of CO, pharmaceuticals, photonics, electronics, optics, biosensors, biomedical and petrochemical industries, and automobiles [2,3,4]. Several approaches are used for synthesizing NPs, such as physical, chemical, and biological methods. Conventional methods of NP production involve two approaches: top-down and bottom-up methods [5]. Generally, physical methods consume enormous energy and dissipate radiation, whereas chemical methods use several toxic chemicals, which are harmful and hazardous to living beings, and release harmful chemicals into the atmosphere. Consequently, recently, researchers have investigated cleaner, greener, scalable, cost effective, and environmentally benign approaches that can avoid toxic chemicals. Environmentally benign conditions require various templates, including microorganisms, algae, fungi, and plants, and small molecules that can act as alternatives for physical and chemical methods. The morphology and size of the NPs can be controlled by methods such as using different concentrations of reducing agent/capping agent, concentrations of precursors, pH, and temperature. Catalytic nanomaterials like platinum, palladium, and cerium display inimitable physicochemical properties and high surface area and have immense potential for application in various fields. platinum NPs (PtNPs) have led to a new revolution in the field of nanotechnology including the chemical industry, automotive sector, and biomedical applications, and therapeutic span. They are also used in numerous biomedical fields including diagnostics with different agents for imaging, medical implants, drug delivery, and photothermal therapy [6,7,8].

PtNPs and their alloys exhibit excellent catalytic properties because of their large surface areas. They are mainly used to reduce pollutants and play a major role in the chemical reactions for synthesis of various chemicals in the chemical industry such as oxidation in the process of organic acid production [9]; dehydrogenation [10,11,12,13]; and hydrogenation reactions for synthesis of biofuel [14], vitamins, and fats [15]. They are also used in green technologies, such as highly polluting aromatic compound [16], solar energy harvesting [17], and water treatment [18,19], and to maintain a clean environmental. Currently, both the research and industrial fields have shown considerable interest in the synthesis of catalytic PtNPs for use in therapeutic applications because of their low toxicity, high stability, and less side effects. Although several reports have studied PtNPs, a comprehensive review about synthesis, characterization, and biomedical applications remains elusive and unexplored [20,21,22,23]. Here, we focus on the synthesis, characterization, and properties of PtNPs and discuss the mechanisms of cytotoxicity and therapeutic applications of PtNPs in cancer cells.

## 2. Classification of Nanomaterials

**Figure 2:**
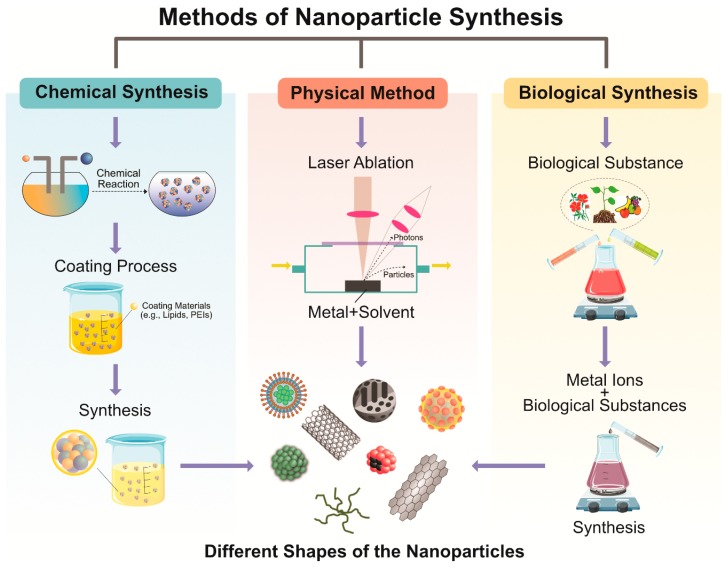
Conventional methods of synthesis of nanoparticles.

NPs are commonly classified based on their morphology, dimensions, shape, composition, uniformity, and agglomeration. Recently, three morphologies of NPs were identified: spherical, crystalline, and flat (Figure 2). NPs are also classified into four types based on electron movement of dimension: 0 dimension; 1D, which includes thin films that are mainly used in electronic devices and sensor mechanisms; 2D, which includes second-generation NPs such as carbon nanotubes, which provide high absorption and stability; and 3D, which includes dendrimers and quantum dots [24,25]. These are further classified based on their chemical structures into inorganic, organic, and carbon-based NPs (fullerenes).

### 2.1. Inorganic Nanoparticles

Inorganic NPs are fabricated from materials without carbon and are further classified into two major categories: metals and metal oxide NPs. NPs are synthesized from metals such as platinum (Pt), silver (Ag), gold (Au), cadmium (Cd), cobalt (Co), iron (Fe), copper (Cu), and zinc (Zn). These NPs have distinctive sizes, shapes, surface areas, and densities.

### 2.2. Metal Oxide Nanoparticles

Metal oxide NP is mainly synthesized for increase their efficiency, reactivity and modify the properties. Aluminum oxide (Al_2_O_3_), cerium oxide (CeO_2_), iron oxide (Fe_2_O_3_), zinc oxide (ZnO), silicon dioxide (SiO_2_), and titanium oxide (TiO_2_) are usually used for synthesis of NPs.

### 2.3. Organic Nanoparticles

Organic NPs are made up of organic polymers such as dendrimers, liposomes, ferritin, and micelles. Micelles and liposomes are sensitive to electromagnetic radiation and the thermal effect [26]. They are mainly used for targeted drug delivery because of their efficient drug delivery capabilities and nontoxicity.

### 2.4. Carbon-based Nanoparticle

Carbon-based NPs are fabricated from carbon and are widely used in biomedical applications. They include fullerenes, graphene, carbon nanotubes, carbon nanofibers, and carbon black.

## 3. Methods for Synthesis of PtNPs

**Figure 3:**
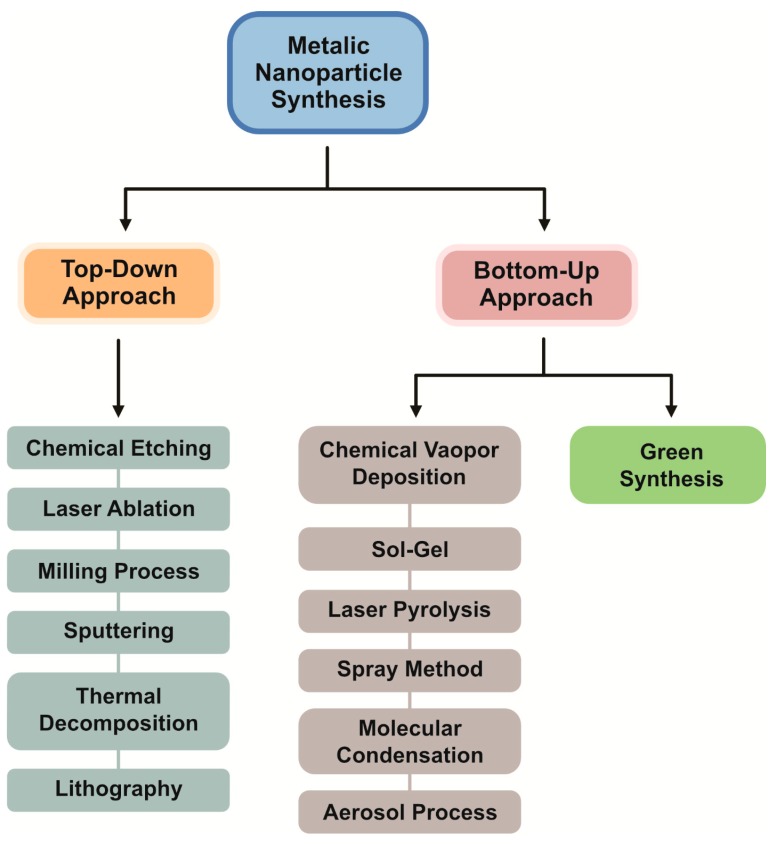
Schematic representation illustrating the various approaches used for synthesis of platinum nanoparticles.

Several methods exist for the fabrication of NPs, which can be largely divided into two major categories: bottom-up and top-down approaches (Figure 3).

### 3.1. Bottom-Up Approaches

To produce PtNPs with controlled sizes and desirable characteristics for various applications, two basic approaches are adopted [27,28]:

(1) Top-down approaches

In the top-down approach, a larger metal is used for the production of NPs by a physical method via the mechanical breakdown of the large metal structure. It is an economic, energy demanding, and lengthy process. Its major advantage is the control of the size distribution and morphologies of NPs [29]. The top-down approach begins with the bulk counterpart that leaches out systematically bit-after-bit, leading to the generation of fine NPs. Several physical methods are adopted for the mass production of NPs such as photolithography, electron beam lithography, milling techniques, anodization, and ion and plasma etching [30].

(2) Bottom-up approaches

**Figure 4:**
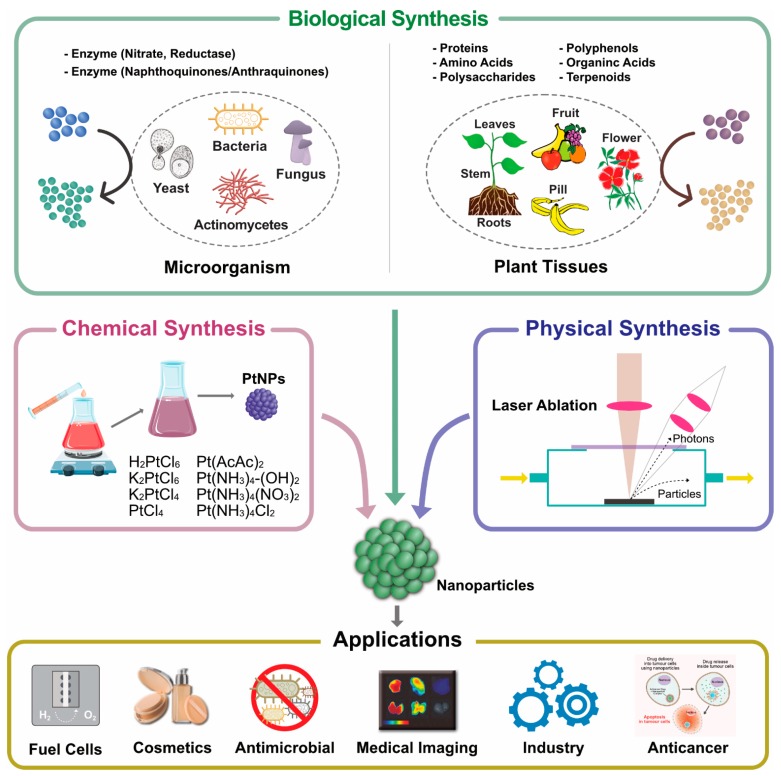
Synthesis of platinum nanoparticles using conventional methods and their applications.

The bottom-up approach involves assembling atoms and molecules to generate a diverse range of NPs. Examples of the bottom-up approach include self-assembly of monomer/polymer molecules, chemical or electrochemical nanostructural precipitation, sol–gel processing, laser pyrolysis, chemical vapor deposition (CVD), plasma or flame spraying synthesis, and bio-assisted synthesis [31]. In general, NP synthesis methods can be divided into three groups—(1) physical methods, (2) chemical methods, and (3) biological methods, also known as constructive methods (Figure 4).

### 3.2. Physical Methods

Synthesis of PtNPs involves the application of mechanical pressure, high energy radiation, thermal energy, or electrical energy to cause material abrasion, melting, evaporation, or condensation to generate NPs. The physical methods of synthesis of PtNPs include evaporation and condensation using a tube furnace at atmospheric pressure. The merits of physical methods are high speed and no use of toxic chemicals, purity, uniform size, and shape, whereas their demerits include less productivity; high cost; exposure to radiation; require high energy, temperature, and pressure; less thermal stability; high amount of waste, high dilution; difficult size and shape tunability; and less possibility in stability. This method is not suitable for preparing familiar shapes and sizes of nanoparticles; it also changes in surface and physicochemical chemistry of nanoparticles. Several physical methods are available, such as laser ablation, arc discharge, vapor deposition, melt mixing, ball milling, sputter deposition, and flame pyrolysis [30]. Laser ablation is a very simple but expensive method and involves the removal of materials from a solid rarely liquids surface by irradiating it with a laser beam. Here, a laser beam is used instead of electric heating. Although the energy efficiency of laser ablation is good, its energy production cost is high. The absorbed laser energy is used by low laser flux materials, thus converting the material to plasma. The major application is weak of agglomeration and lack impurities, previously several researchers reported that preparation of PtNPs by pulsed laser ablation in liquid (PLAL) [31,32,33,34]. Milling process involves reducing the size of particles and blending the particles into new phases. Materials can be selectively removed from the substrate in a suitable medium in the presence of reducing agents. C_6_H_5_O_7_Na_3_, N_2_H_4_, and NaBH_4_ are frequently used for chemical reduction processes. Solvothermal processes increase the solubility of the reactants and enable the reaction to take place at a low temperature. It is a low-temperature method that uses polar solvents, which are used under pressure and at above their boiling points. Inert gas condensation (IGC) methods involve evaporation of metals by vacuum chambers filled with inert gas at a typical pressure of 100 Pa. IGC is a highly efficient method for the synthesis of good quality silver and PtNPs [35]. In this method, inter atomic collision occurs between gas atoms within a chamber, and the evaporated metal atoms lose their kinetic energy to condense in the form of a crystal which accumulates in liquid nitrogen. This method is mainly used for synthesis of gold NPs.

### 3.3. Chemical Methods

Chemical synthesis of NPs follows the bottom-up approach. This process mainly involves the use of water-soluble cations as a precursor to trigger their reduction to metal monomers, and the process is called nucleation. The growth of particles where it assembled cluster of reduced metal atom automatically stop the growth controlled by reducing agent/capping. The particles reached a certain size which is stable thermally. Nanomaterials are synthesized by the interaction of atoms and smaller molecules. Various chemical synthesis techniques include the sol–gel process, pyrolysis, CVD, microemulsion, hydrothermal, polyol synthesis, and plasma-enhanced chemical vapor deposition. Chemical preparation involves synthesis of metal NPs in a chemical solution, and various chemical reactions and chemical compositions are used for these purposes. For instance, the chemical reduction of metal ions inside reversed micelles in a nonpolar solvent is the most commonly employed method for the preparation of metal nanoparticles (MNPs) [36]. For instance, first, a metal slat dissolved in water is confined in the reversed micelles and is reduced into MNPs by chemical reduction. Size control of the particles is important and is regulated by volume of reversed micelles and ratio of water. Several points need to be considered for synthesis of PtNPs such as the appropriate shape and size and the appropriate selection of solvent, temperature, and reducing/capping agent. The method should also avoid aggregation. The advantage of chemical methods is cost effective, high versatility in surface chemistry, easy functionalization, high yield, size controlled, thermally stable, and reduced dispersity. Disadvantages of chemical methods include low purity and the use of toxic chemicals and organic solvents, which can be hazardous to human beings and the environment.

**Figure 5:**
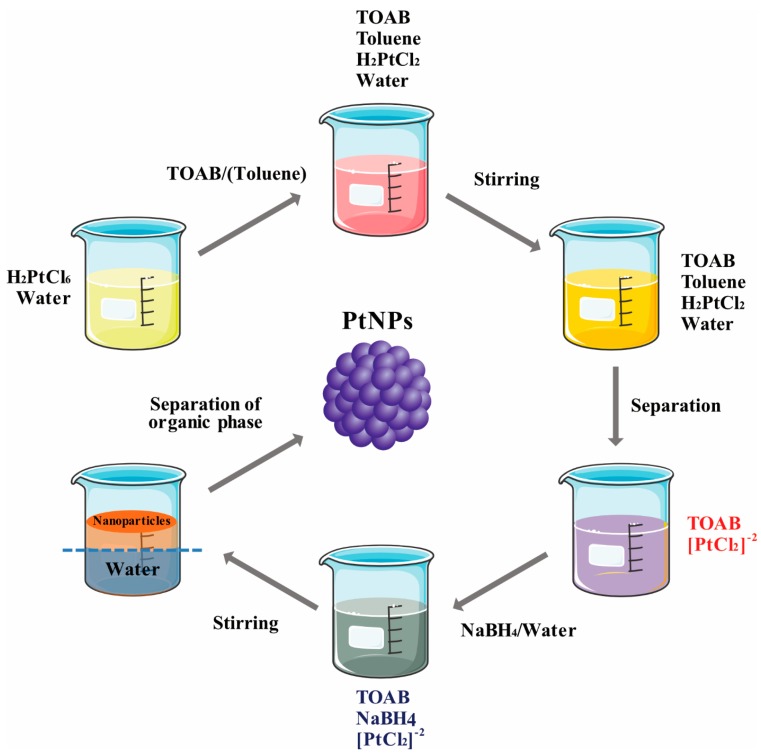
Chemical reduction for platinum nanoparticle synthesis.

**Figure 6:**
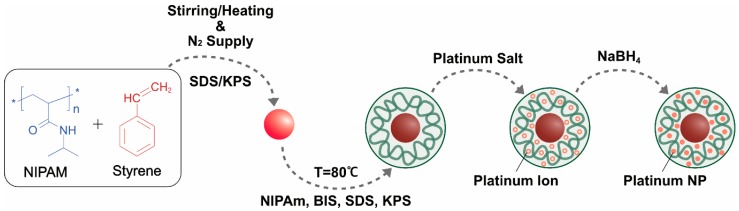
Chemical synthesis of doped platinum nanoparticles.

Chemical methods are important, established methods of NP synthesis. In the early 1920s, Adam et al. [37] prepared bulk type PtO_2_ using a fusion method at 450 °C. Next, numerous methods were used to modify the material into NPs. NP production is efficient and low cost but requires toxic chemicals. Several studies have reported chemical synthesis of PtNPs [38,39,40,41,42,43,44]. There are three imperative components employed for the synthesis—metal precursors, capping/stabilizing agent, and reducing agent. In general, PtNPs can be synthesized by two methods—the top-down and bottom-up processes. Numerous chemical methods have been used for synthesis of NPs such as wet chemical reduction [45], microemulsion [46], electrochemical process [47,48], confined reaction, photochemical reduction [49,50], hydrolysis [51], thermal decomposition [52], sono-decomposition [53,54], chemical vapor deposition [55,56], and coprecipitation. Wet chemical reduction is often used in laboratory research to control particle sizes. Chemical reduction is mainly used for colloidal metal NP production, in which chemical agents reduce the metallic ion, thus forming metallic NPs (Figure 5). Reducing agents like sodium borohydride (NaBH4), potassium bitartrate (KC_4_H_5_O_6_), methoxy polyethylene glycol (CH_3_O(CH_2_CH_2_O)nH), trisodium citrate dihydrate (Na_3_C_6_H_9_O_9_), ascorbate, and elemental hydrogen are used for the reduction process. The size and shape of the synthesized NPs varies depending on reaction temperature [57], reducing agent [58,59,60,61], and concentration of the platinum compound (Figure 6).

### 3.4. Biological Synthesis of PtNPs

**Figure 7:**
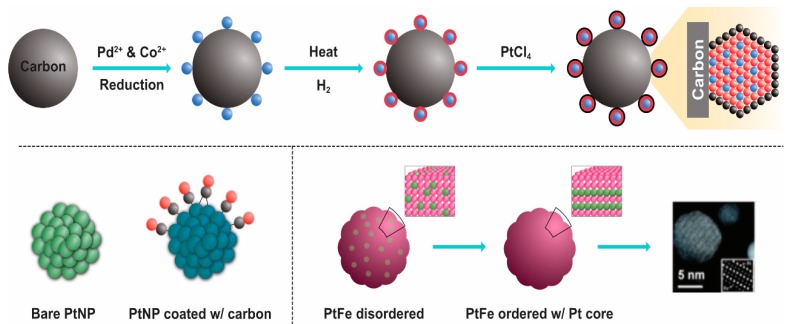
Synthesis of platinum nanoparticles using various types of metal salts.

Biological synthesis or biomolecule-assisted synthesis is commonly used for the production/fabrication of PtNPs. The advantage of biological methods is that they are simple, facile, and environmentally friendly, and the synthesized nanomaterials are nontoxic and biocompatible. NPs with definite size and shape can be produced by adjusting the concentration of reducing agent, temperature, and pH [62]. The synthesis of PtNPs using biological systems is limited compared with the synthesis of silver, gold, and other metal NPs. Therefore, we critically assess the role of biological systems in the synthesis of PtNPs. A few plant species are used for the synthesis of PtNPs including *Azadirachta indica*, *Diospyros kaki*, *Ocimum sanctum*, and *Pinus resinosa*. Several studies have reported the biological synthesis of PtNPs using different microorganisms. For the synthesis of PtNPs, water soluble metal salts are frequently used, such as H_2_PtCl_6_, K_2_PtCl_6_, K_2_PtCl_4_, PtCl_2_, Pt(AcAc)_2_, Pt(NH_3_)_4_-(OH)_2_, Pt(NH_3_)_4_(NO_3_)_2_, and Pt(NH_3_)_4_Cl_2_ (Figure 7). The potential benefit of biological method of synthesis was that the produced nanoparticles were soluble, biocompatible, sustainable, chemical free, cost effective, and eco-friendly. The disadvantage of this method is hard to control shape, size, crystal growth, stability and aggregation, and possible endotoxin and time-consuming purification processes.

Several methods have used for the synthesis of NPs from various sources and diverse methods. Each method has certain limitations and advantages. Stable NPs are prepared from chemical methods, but they are unconducive for biomedical applications, especially for drug delivery systems. Chemical methods lead to concerns regarding biosafety, drug activity, and clinical application. Therefore, it is imperative to develop suitable methods for the synthesis of NPs from biological systems, which are facile and versatile. The fabrication of PtNPs with morphological characteristics, such as nanotubes, cubic NPs, and nanoclusters, is possible and has attained considerable interest for application in catalysis [63,64,65]. Therefore, clean, nontoxic, environmentally friendly, and green chemistry approach-based synthesis of NPs is in great demand. Currently, researchers are exploring biological systems including bacteria-, fungi-, plant-, and small biomolecule-based synthesis of NP. Biological systems are considered the potentially environment friendly nanofactories. These methods have been suggested as alternatives to physical and chemical methods, and the use of alternative methods offers numerous advantages such as no toxicity, cost effectiveness, rapid synthesis, robotic, environmentally benign, monodisperse, and large-scale production, reducing waste production and decreasing production cost [66]. Such methods also have immense potential for application because the sizes of particles produced are very small.

### 3.5. Synthesis of Platinum Nanoparticle Using Bacteria

**Figure 8:**
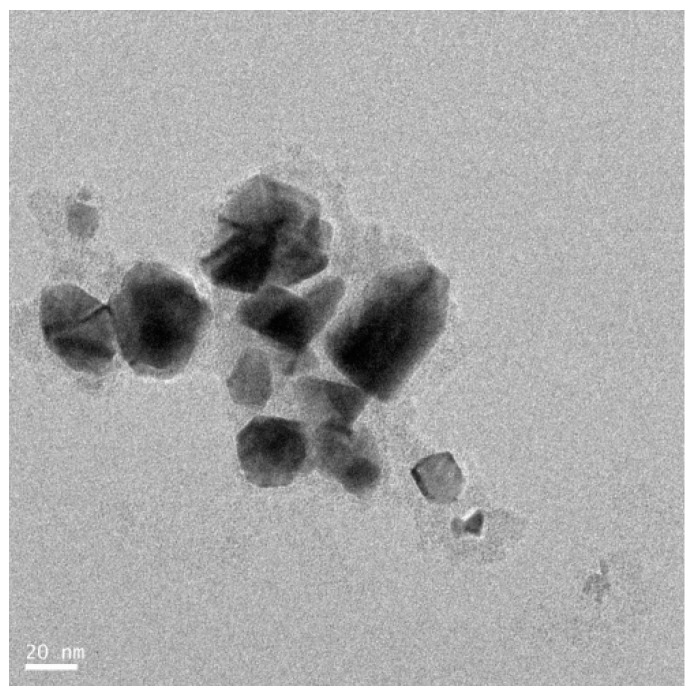
Transmission electron microscopy image of platinum nanoparticles produced by *Bacillus* spp.

Single cellular and multicellular organisms are known to produce inorganic material either extracellularly or intracellularly [67]. Generally, bacteria produce NPs by the reduction process. The conversion of metal ion to NP using intracellular signaling pathways involves bacterial enzymes (Figure 8). The major advantages of bacteria-based NP synthesis is the ease of handling. The synthesis can also be easily modified using genetic engineering techniques for specific purposes, to reduce toxicity, and to obtain sustainable NP production. This method also has some disadvantages like laborious method, high cost, downstream processing, and less control over their size and shape. For instance, metal NPs like silver were synthesized using both intra- and extracellular bacterial extracts [68,69]. This process consumes a lot of time for downstream processing for the purification of AgNPs from cellular extracts. Riddin et al. [70] reported the successful synthesis of geometric PtNPs using cell-free, cell-soluble protein extracts from a consortium of sulfate-reducing bacteria compared to whole cells from the same culture, which produce amorphous Pt^(0)^. Synthesis of PtNPs was carried out by sulfate reducing bacteria *Desulfovibrio desulfuricans* [71] and *Acinetobacter calcoaceticus* [72]. These bacteria can potentially reduce platinum (IV) ion into platinum^(0)^ NPs within 24 h, and the maximum production was observed at pH 7.0 under 30 °C. The NPs are 2–3.5 nm in size with a cuboidal structure. PtNPs are deposited by metal-ion reducing bacterium *Shewanella algae*, and the resting cells of PtNPs by reducing ion PtCl_6_^2−^ into elemental platinum at neutral pH and room temperature. NPs 5 nm in size have been reported to be located in the periplasmic space of *S. algae.* The biological process involves two main processes—uptake and deposition or assimilation [73].

### 3.6. Synthesis of Platinum Nanoparticles Using Fungi

**Figure 9:**
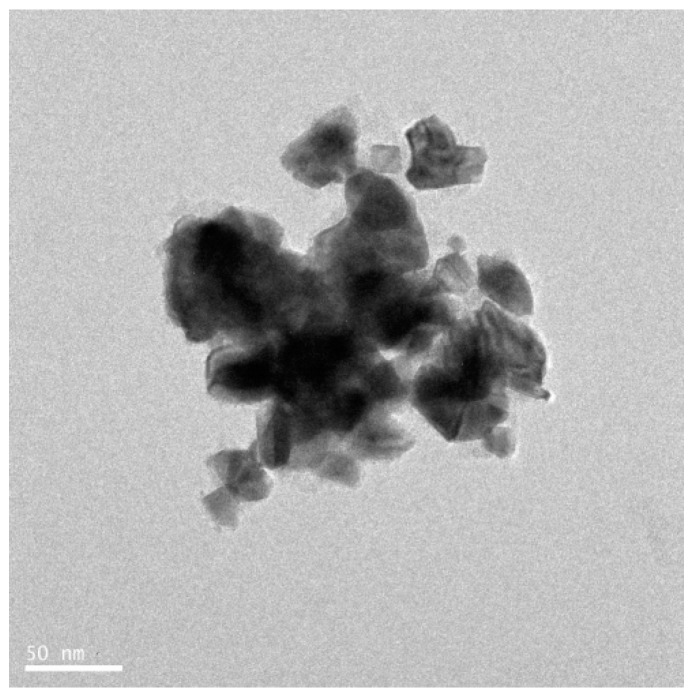
HR-TEM micrograph of platinum nanoparticles produced by fungi (*Ganoderma* spp.).

Numerous fungal species have been used for synthesis of NP. The use of fungi, as compared to prokaryotes or plants, is more advantageous because monodispersed NPs with well-defined dimensions are produced, fungi require simple media for growth, scale-up production and downstream processing are easy, the biomass is easy to handle, high amounts of proteins are secreted [74,75,76], enzyme production enhances the reductive properties and also increases the amount of NP produced [77], very stable NPs are produced, and molecular aggregation can be prevented [78,79]. Thus, researchers have explored the use is fungus as an excellent candidate for the fabrication of nanomaterials. Most fungi produce metal NPs either by intracellular or extracellular processes. Extracellularly produced NPs have good commercial feasibility and are nontoxic. Syed and Ahmad [76] reported that the synthesis of PtNPs using *Fusarium oxysporum*, which produces PtNPs extracellularly at room temperature. The morphology and size of the NPs was found to be spherical and 15–30 nm, respectively, as determined by TEM analysis (Figure 9). Castro-Longoria et al. [80] reported the use of *Neurospora crassa* fungus for the synthesis of PtNPs. They produced NPs intracellularly at an ambient temperature. The produced particles were found to be quasi-spherical and single crystalline nanoaggregates with an average size between 20 and 110 nm. Altogether, these studies confirmed that fungal extracts can be used as a reducing and stabilizing agent for synthesis of PtNPs.

### 3.7. Green Synthesis of Platinum Nanoparticles Using Plants

**Figure 10:**
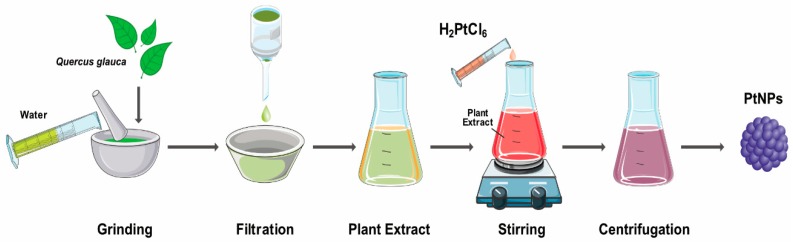
Platinum nanoparticles synthesized by plant extract/phytochemical method.

Common biological methods for synthesis of NPs include several organisms such as bacteria, actinomycetes, algae, and fungi. Although microorganisms are exploited for the synthesis of PtNPs, controversy still exists regarding the use of microorganisms because the production time of NPs is high because of the time required to grow bacterial/fungus cultures and for bacterial cell maintenance. Therefore, researchers are interested in exploiting the use of plants and plant extracts, which are readily available and abundant and do not require any media to grow. Plant-based synthesis of NPs has numerous advantages over the other types of biological methods (Figure 10). Gardea-Torresday et al. [81] first synthesized NPs in living plants and fabricated gold NPs from Alfalfa seedlings with size ranging from 2 to 20 nm. Biological templates used for the synthesis of PtNP are shown in Table A1.

The extracellular synthesis of PtNPs in the plant system was first described by Song et al. [82]. The *Diospyros kaki* leaf extract was used for the synthesis of NPs. At 95 °C, color changes were observed due to the excitation of surface plasmon vibration in the metal NPs, which was analyzed by UV–Vis spectroscopy; the conversion of platinum was observed at 477 nm. The TEM studies indicated the formation of NPs with an average size of 2–12 nm. The leaf extract was used as a reducing agent, and it was an extracellular and non-enzyme-mediated process. They used low biomass concentration, and high yield was achieved. Production of pentagonal and hexagonal shapes of the PtNPs was accomplished using an extract of *Fumariae herba* [83]. The synthesis was carried out at 50 °C for 4 h. Color changes were observed from yellow to brown and UV–Vis spectrometer analysis showed the peak. Both results confirmed the formation and complete reduction of Pt^4^ ions to Pt^(0)^ NPs. The average size of NPs was 10–30 nm. The catalytic activity was analyzed by studying the reduction of two different dyes-methylene blue and crystal violet. Sheny et al. [84] reported the synthesis of PtNPs from *Anacardium occidentale* leaf extract. Different pH values were used for the synthesis of NPs. Qualitative analysis of color, which is a characteristic of PtNP formation, and UV analysis revealed the reduction of platinum ions to PtNPs; the pH range of 6 to 9 was suitable for the formation of NPs. TEM micrographs showed the formation of crystalline NPs of irregular rod shapes. NPs have good potential for the reduction of aromatic nitrocompounds because secondary metabolites are present in the leaf. NanosolutionS-containing small amounts of PtNPs are found to have high thermal conductivity than those without NP solutions. Therefore, the thermal conductivity of platinum colloid shows comparative to increase with volume of fraction. PtNPs were also synthesized neem leaf extracts [85]. The color changes from yellow to brown and UV–Vis spectrum analysis suggested the formation of PtNPs and the reduction of ions to platinum. The TEM images showed the formation of polydispersed small-to-large spherical NPs with a size ranging between 5 and 50 nm. FTIR spectra showed sharp peaks corresponding to carbonyl, alkynes, and aliphatic amines. The extract contained terpenoids which act as a reducing and stabilizing agent. Piper beetle leaf extract has been used to synthesize PtNPs [86]. The TEM images show a cubic (fcc) structure and monocrystalline nature. The average size of the NPs was 2 nm. The fcc structure of NPs clearly indicates that the NPs were composed of highly crystalline PtNPs. *Dioscorea bulbifera* tuber extract has been used for the synthesis of PtNPs. The reaction conditions were 95 °C for 5 h, and the morphology of the synthesized NPs was found to be spherical and 5 nm in size, as analyzed by TEM analysis.

**Figure 11:**
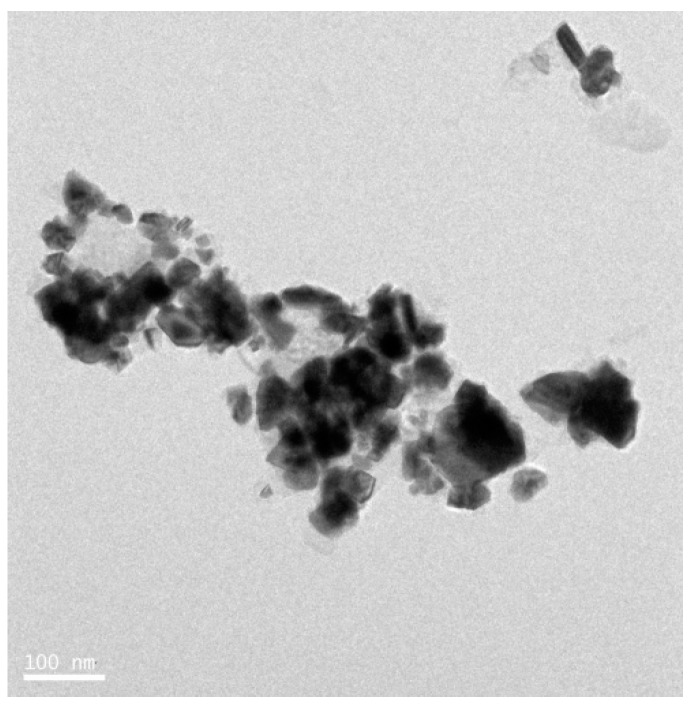
HR-TEM image of platinum nanoparticle produced by plant extracts of *Ipomea carnea*.

A study described the biogenic fabrication of PtNPs using the leaf extract of medicinal plant Ocimum sanctum [87]. The optimal reaction temperature was set at 100 °C, which is higher than that required for normal synthesis processes. A high temperature is favorable for rapid synthesis of NPs. By this process, the authors produced NPs with an average size of 23 nm, and the shapes of the particles were irregular. The leaf extracts are bioactive, containing various antioxidants, phenolic compounds, flavonoids, ascorbic acid, gallic acid, terpenoids, amino acid, and certain proteins that act as reducing agents. Previously, Huang et al. [88] demonstrated that biomolecules derived from plant extracts that are suitable reducing agents are found to have a major role, over their counterparts, as protecting agents. For instance, *Ipomea carnea* plant extracts produce NPs with an average size of 50 nm (Figure 11).

*Gloriosa superba* roots are mainly used as a germicide and for treatment of leprosy, inflammation, flatulence, intermittent fever, debility, ulcers, piles, hemorrhoids, scrofula, dyspepsia, worm infestation, arthritis, and against snake poison [89]. The fabrication of PtNPs at high temperatures formed spherical particles 0.83 nm in size. This result agreed with the production of NPs with other medicinal plants like *D. bulbifera*, *Barleria prionitis*, and Ocimum. These results were finding to synthesis of PtNPs by using medicinal plants. Similar sizes of PtNPs were produced by *Cacumen platycladi* at 90 °C, ranging from 0.8 to 2 nm. Coccia et al. [90] reported the one-spot synthesis of PtNPs from natural second biopolymer lignin isolated from red pine (*Pinus resinosa*) and fulvic acid.

**Figure 12:**
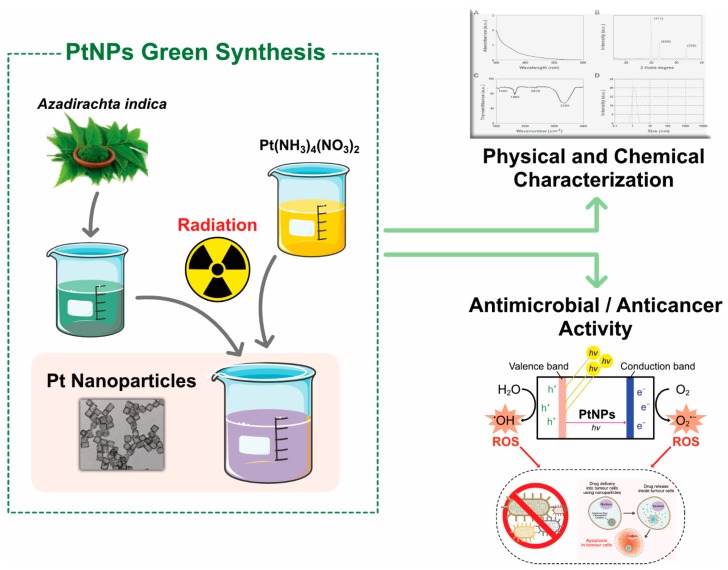
Synthesis, characterization, and application of platinum nanoparticles produced by plant *Azadirachta indica.*

The reaction was carried out at optimum pH (7.0) and at 80 °C. The formation of NPs with lignin was confirmed by color changes from orange to dark brown, as analyzed by the subsequent UV spectra analysis at 257 nm after 4 h. Peaks were also obtained for fulvic acid at 280 nm in the presence of phenolic groups. NMR spectral analysis showed two signals (PtCl_6_^2−^ and PtCl_5_ (H2O)^−^ which gradually disappeared as a result of the formation of NPs. The shape of the platinum particle was predominantly irregular forming clusters and particles ranging in size from 6 to 8 nm. The resultant lignin and fulvic acid were found to act as both reducing and stabilizing agents. Najlaa S. Al-Radadi [91] used date extracts to biologically synthesize PtNPs. The ensuing size of the PtNPs ranged between 1.3 and 6 nm, and the shape of the NPs was homogeneous small spheres. The flavanols worked as reducing and capping agents. Assessment of the resulting PtNP solution demonstrated the antibacterial activity against *E. coli* and *B.* *subtilis*, providing promising results against several cell lines. Xiaobo Lin et al. [92] produced spherical and cubic NPs and spherical platinum nanoclusters from natural wood. The synthesized NPs were highly stable, and the catalytic reduction of *p*-nitrophenol was promising as a one-step process. The concentration of the precursors and pH played a major role in the controlled fabrication of PtNPs. Leaf extracts from invasive weed Lantana (*Lantana camara* L.) was used for the fabrication of PtNPs. The reaction conditions were 95 °C for 8 min, and the color was changed to black, indicating the formation of NPs. The UV–Vis spectra revealed the reduction of platinum ions. The TEM images showed that the NPs were spherical in shape and 35 nm in size [93]. For example, *Azadirachta indica* is potentially used for synthesis of various nanoparticles silver, gold, palladium, and platinum, and synthesized particles were characterized by various analytical techniques (Figure 12).

*Prunus yedoensis* tree gum extract was found to produce spherical shaped PtNPs with an average particle size of 10–50 nm [94]. The synthesized NPs were found to be effective against pathogenic fungi. The tea polyphenol from *Camellia sinensis* was used for fabrication of PtNPs and acted as the reducing and stabilizing agent. PtNPs obtained from tea polyphenol showed a flower-shaped morphology with sizes ranging from 30 to 60 nm. The inhibition of cell proliferation was also evaluated, and it was found to induce apoptosis of cervical cancer cells [95]. Recently, spherical PtNPs of 5–150 nm in size were synthesized from the coral vine *Antigonon leptopus*. The conversion and formation of PtNPs was confirmed by rapid color changes at an elevated temperature of 95 °C, which represented the formation of PtNPs. The *Antigonon leptopus* extract acted as a stabilizing and reducing agent for the synthesis of PtNPs [96]. PtNPs have been synthesized using leaf extract from *Barleria prionitis*. The color change from light brown to dark brown at 100 °C indicated the formation of PtNP, and the synthesis was confirmed by UV–Vis spectral analysis. Monodispersed NPs of 1–2 nm in size were synthesized using *B. prionitis* extract, and the FTIR spectra showed several functional group constituents of *B. prionitis* leaf, which is mainly involved in the reduction and stabilization of NPs. The synthesized PtNPs showed cytotoxic effects against the breast cancer cell line MCF7 [97]. Previously, Preeti Dauthal et al. [98] reported the fabrication of PtNPs from agroindustrial waste of *Punica granatum* peel extract. TEM images showed that the particles were spherical with sizes ranging between 16 and 23 nm, and X-ray diffraction (XRD) patterns suggested the synthesis of powder form of PtNPs in the plane in predominant orientation. FTIR peaks were observed at 3424 cm^−1^, which shifted to 3439 cm^−1^, suggesting that the phenolic group was present in the peel extract, which is involved in the reduction and stabilization of NPs. PtNPs with a spherical shape and an average size of 20.12 nm were synthesized from pomegranate extract, and the reaction was carried out at room temperature for 24 h. The formation of NPs was analyzed by visualization of color changes from yellow to brown, and subsequently, synthesis was confirmed by UV–Vis spectral analysis.

### 3.8. Synthesis of Platinum Nanoparticles Using Purified Plant Compounds

**Figure 13:**
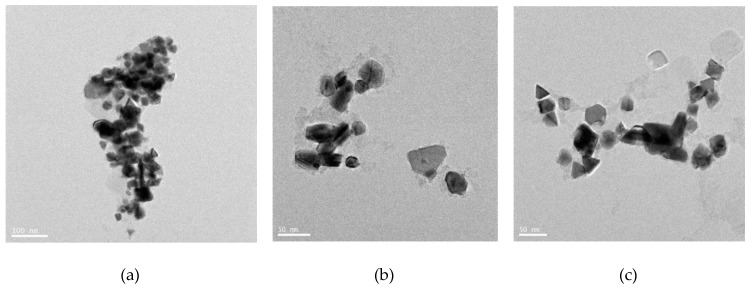
High-resolution TEM images of platinum nanoparticles and their different shapes and sizes produced by various secondary metabolites of plants. Left panel (**a**): Platinum nanoparticle synthesis assisted by terpenes. Middle panel (**b**): Platinum nanoparticle synthesis assisted by phenolic compounds. Right panel (**c**): Platinum nanoparticle synthesis assisted by S-containing compounds.

Generally, plant extracts are used for the synthesis of metallic NPs. However, there is no clear understanding regarding the mechanism of synthesis of NPs. Therefore, studies are necessary to understand the synthesis of NPs using individual purified compounds. In addition, to avoid downstream processing, reduce time for production, and increase the scale-up processes, several studies reported the synthesis of various metallic NPs using purified phenolic compounds such as a conjugation of sugars, secondary metabolites, and proteins [99]. TEM images shows the images of purified plant compound synthesized PtNPs exhibited various shapes, such as spherical, cubic, rectangle, triangle, octahedral, and tetrahedral or truncated cubic, hexagonal, octahedral, and tetrahedral, indicating their very sharp corners, edges, and facets (Figure 13). Synthesized NPs revealed potent anticancer activity against a human breast cancer cell line [100]. Similarly, biologically synthesized PtNPs exhibited potential cytotoxicity against various human cells such as human monocytic cells and human bone OS epithelial cells [101,102]. All these data provided strong evidence for the synthesis of PtNPs using natural products and its potential anticancer activity.

## 4. Characterization of PtNPs Using Various Analytical Techniques

Generally, the synthesized NPs are characterized by various analytical techniques such as UV–Visible spectroscopy, XRD, energy-dispersive spectroscopy (EDS), Fourier transform infrared (FTIR) spectroscopy, nanoscale infrared spectroscopy, dynamic light scattering, scanning electron microscopy, TEM, atomic force microscopy (AFM), and extended X-ray absorption fine structure spectroscopy (EXAFS). Briefly, here, we describe the principle of each instrument and how it is useful for NP detection.

### 4.1. UV–Visible Spectroscopy

UV–Visible spectroscopy is generally used to confirm the synthesis and stability of metal NPs/colloidal particles. Synthesis is confirmed based on the absorbance of the samples at wavelength from 230 to 800 nm, and NPs ranging from 1 to 100 nm can be used for analysis. The PtNP electron and valence bands are very close to the each other. The free electrons give rise to the surface plasmon resonance absorption band [103]. UV absorption spectroscopy is not only used for the confirmation of synthesis, but also used for the quantitative determination of particles in colloidal solutions. Furthermore, absorption spectra determine the size of the particles and can be used for quantitative and qualitative analysis of particles. Spectroscopy involves the measurement and interpretation of electromagnetic radiation absorbed or emitted when the molecules, atoms, or ions of a sample move from one energy state to another. NP samples are analyzed using the absorption of ultraviolet light (200–400 nm) by the molecule which results in the excitation of the electrons from the ground state to higher energy state. The salient features of UV–Vis spectroscopy are easy handling, sealed optics, double choppers, and the spectral bandwidth, which can be set to 0.2 nm.

### 4.2. Fourier Transform Infrared Spectroscopy

Fourier transform infrared spectroscopy (FTIR) is considered a powerful and simple technique. It has an imperative role in biological systems for measuring the concentration of chemicals, surface chemistry, functional group, and atomic arrangement of the biological NP samples [104]. In NP synthesis, FTIR can analyze whether the biomolecules are involved in synthesis or not. It also shows what biomolecules are present in the sample [105,106,107,108]. FTIR measurement depends on the vibration of molecular bonds positioned at various frequencies and the type of bonds. The salient features of FTIR are high sensitivity, high cube corner interferometer, customizable workspaces, and hyperspectral imaging.

### 4.3. Nanoscale Infrared Spectroscopy

Infrared spectroscopy (IR) involves a reproducible instrument which is used to analyze the structure of matter at the molecular scale based on the resonant vibration modes of various molecules [109]. It can unveil the elemental composition and bonding arrangement. The intensity of radiation and frequency of the spectrum are plotted, and a unique spectrograph is used to compare with international standards and identify the molecules. Infrared spectroscopy provides the ability to characterize and identify chemical species. However, it its resolution is restricted in the order of 5–10 µm. NanoIR is a probe-based measurement tool used to measure the chemical composition of samples at the nanoscale. It comprises key elements of both infrared spectroscopy and AFM to enable the acquisition of infrared spectra at spatial resolutions of 50–200 nm. The salient features of nanoIR include multifunctional measurements. This instrument provides a complete picture of samples by integrating topographic, spectroscopic, mechanical, and thermal properties of samples.

### 4.4. Dynamic Light Scattering

Dynamic light scattering (DLS), also called photon correlation spectroscopy and elastic light scattering [110,111], involves a table top instrument and is an easy-to-handle technique. Furthermore, the method is accurate, less time consuming, inexpensive, and data reproducible and allows analysis of high-molecular-weight samples. It is mainly used to evaluate the size and surface charge of NPs. Colloidal dispersed NPs can only be measured by Brownian motion [112]. It also has certain limitations such as aggregation and is not suitable for the analysis of nonspherical nanomaterials with heterogeneous size distributions. The working principle of the DLS involves screening the elastic scattering intensity of light from the Brownian motion of the sample. The particle size can be obtained from the motion-dependent auto correlation function of Einstein equation [113,114]. The salient features of DLS include measurement of samples ranging in size from 0.3 nm to 6 µm and a minimum volume 10 µL of sample suspension with an accuracy of ±2% and a precision of ±1%. It can measure the zeta potential of colloidal, nanoparticulate, and macromolecular samples in the size range of 1 nm to 100 µm with a minimum volume of 175 µL. It is frequently used to probe the behavior of complex fluids such as concentrated polymer solutions. It can be used to analyze a wide range of suspension concentrations (from 0.1 ppm to 40% v/v) depending on the sample type.

### 4.5. Electrophoretic Light Scattering (ELS)

Electrophoretic light scattering (ELS) is a technique used to measure the electrophoretic mobility of particles in dispersion, or molecules in solution. This mobility is often converted to Zeta potential to enable comparison of materials under different experimental conditions. The basic principle of this instrument is electrophoresis, which is mainly based on electric charges, when electric field is applied into the dispersion, particles, or molecules are having net zeta potential will migrate towards the oppositely charged electrode with a velocity, known as the mobility, that is related to their zeta potential. Malvern Instruments offers equipment to measure the electrophoretic mobility of particles using electrophoretic light scattering. For example, The Zetasizer Nano provides a simple, fast, easy, and accurate way to measure zeta potential and free from cross contamination due to use of unique disposable capillary cell.

### 4.6. XRD

XRD is one of the best methods for characterization of the crystalline form of organic and inorganic materials. Particularly, the cubic and crystalline nature and purity of PtNPs can be measured. XRD is nondestructive, simple, highly sensitive, depth profile, table make, and user friendly. It also contains several application aspects such as pharmaceutical, glass, polymer, geological, and forensic and has been used to analyze the chemical composition by quantitative and qualitative measurements by measuring the degree of crystallinity and providing accurate information on the atomic arrangements at interfaces. The crystal structure describes the atomic arrangement, position, and intensity of the diffraction peaks. The wavelength of X-rays is on the atomic scale. Therefore, it is mainly used for probing the structure of nanomaterials. A single beam of X-rays is scattered by each atom in the powder sample. The scattered beams reflected by any crystal form many diffraction patterns. Where the X-rays scattered sample the maximum intensity of the peak at a particular angle. This peak reflects the structural and physicochemical characters of the crystal. The working principle of XRD follows Bragg’s Law [115,116]. The unique pattern of the diffraction beam is compared with the reference database in the Joint Committee on Powder Diffraction Standards (JCPDS). Elemental composition of metal NPs is usually analyzed by EDS [117].

### 4.7. Scanning Electron Microscopy (SEM)

SEM is a versatile, nondestructive analytical method that involves a microscope with a large specimen chamber, with a working distance of 8.5 mm, owing to a combination of inclined detectors and the sharp conical objective lens. SEM is used for surface and dimensional measurements of nano and micro structure analysis of samples and is a type of imaging technique. It is a valuable tool for the evaluation of material structure. It is fast and easy to operate and provides reliable data. In addition, SEM is applied in numerous fields such as biological science, biomolecules, biomedical fields, and material sciences. In biomedical sciences, it is primarily used to characterize cells and organ and tissues surfaces based on 3D images. It provides more information about the cell surfaces. The working principle of SEM is based on generation of electron beams that have magnetic properties. Their magnetic field interacts with the sample to produce secondary and backscattered electrons, which are used for detection [118,119,120,121]. The detection of transmitted electrons is very useful to study nanomaterials. Furthermore, the elemental composition and concentration of samples can be analyzed using SEM-EDX.

### 4.8. TEM

TEM is a common method and an indispensable tool for the characterization of NPs. This main advantage is the determination of the morphology, crystal structure, and size and the qualitative and quantitative analysis of prepared NPs and internalized NPs in cells or tissues [122,123,124,125]. Conventional TEM techniques are used to analyze the sample ubiquitously. However, this technique has certain limitations related to samples >300 nm thick, only limited areas of which are screened; for particle size >100 nm, low magnification is achieved; TEM cannot characterize small-sized NPs (10–20 nm or less than 10 nm); and sample preparation is destructive. TEM is classified based on applications like immune and energy filtered electron microscopy.

### 4.9. AFM

AFM is also called scanning force microscopy (SFM). It involves a small, powerful, high-speed, benchtop, and compact instrument. It is a potential tool for image analysis of biological samples on the nanometer scale; particularly, it is used to measure surface thickness of nanomaterials. This instrument works based on the interaction forces between the surface of the sample and tip of the cantilever. It has numerous applications in various fields divided into two categories: imaging and non-imaging. Three decades ago, AFM techniques were used for lipid membrane analysis [126]. AFM is also used to analyze biological samples and cascade–DNA images [127]. A major limitation of AFM is sample preparation for the study of biological sample. AFM is a type of scanning probe microscopy (SPM). The AFM tip contacts the surface of the sample, and lower and higher frictional forces are measured in this mode [128]. It is an innovative tool used in various fields such as nanotechnology, physics, chemistry, biology, and medicine. SPM is mainly used to construct 3D maps with nanostructural details of biological molecules, cellular components, and cells surfaces [129]. It is also used to characterize mechanical properties of cell membranes [130] and analyze viscoelasticity [131], stiffness of the cell membrane [132], cell adhesion [133], living cell [134], gene packaging material [135,136], mRNA expression [137], and immunological studies [138]. There are two types of SPM: scanning tunneling microscopy (STM) and near field scanning optical microscope (NSOM). STM is used in the industrial sector and for basic research to obtain atomic scale images of surfaces and to analyze the surface defects, molecule size, and aggregation to the surface. It works based on quantum mechanics and piezoelectric effects. NSOM is another type of SPM which uses a wavelength light source used as a scanning probe. This probe is used for scanning. It scans over the surface of material at a height of a few nanometers [139].

### 4.10. EXAFS

EXAFS is used to analyze the structural information of a sample, which is based on the X-ray absorption spectrum, and is mainly used for elemental analysis of inter atomic distance and structural disorders [140] as well as for other applications such as in natural science, biological science, and earth science. Furthermore, in contrast to other diffraction techniques, samples in all forms can be analyzed, such as solid, liquid, glass, gas, and crystalline samples. This flexibility is the main advantage for biological science analysis. Kossel [141] recorded the X-ray absorption spectrum using conventional and the se of synchrotron radiation as a source of high intensity of X-ray. It enhances the quality of data, but is time-consuming.

## 5. Multifarious Applications of PtNPs

**Figure 14:**
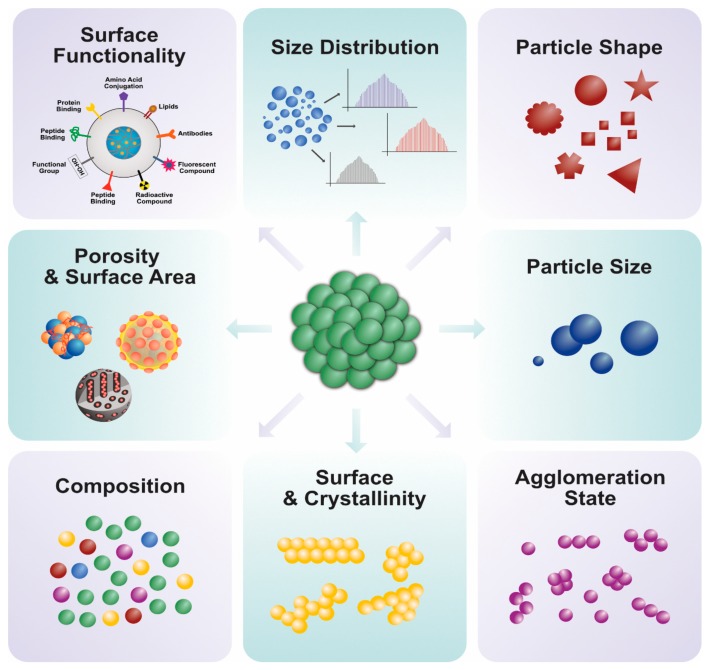
Physicochemical properties of platinum nanoparticles.

The unique features of PtNPs, such as surface functionalities; size; size distribution; shape; porosity; surface area; composition; crystalline nature; agglomeration; and electro, catalytic, thermal, and plasmonic properties, make their application desirable in various fields (Figure 14). Recently, PtNPs have garnered a steadily growing interest for different biomedical applications such as antimicrobial agents, anticancer agents, targeted drug delivery, hyperthermia, photoablation therapy, bioimaging, and biosensing. Bimetallic NPs such as iron platinum (Fe–Pt) NPs possess unique chemical and magnetic properties such as chemical stability, superparamagnetization, high Curie temperature, high saturation magnetization, and high X-ray absorption. These unique properties provide the potential of their application in hyperthermia treatment, as MRI contrast agents, in drug delivery, and as biosensors [142,143,144,145,146,147,148,149,150,151,152,153,154,155,156,157,158,159].

## 6. Antibacterial Activity of Platinum Nanoparticle

**Figure 15:**
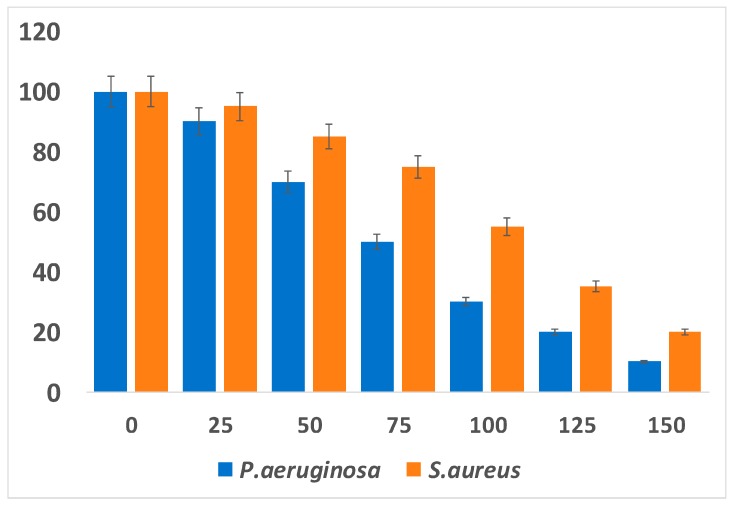
Antibacterial activity of PtNPs against Gram negative and Gram-positive bacteria. Effect of apigenin mediated synthesis of PtNPs on cell survival of *P. aeruginosa* and *S. aureus* was analyzed. All the test strains were incubated in the presence of different concentrations of PtNPs (25–150 µg/mL). Bacterial survival was determined at 24 h by a colony forming unit (CFU) assay. The results are expressed as the means ± SD of three separate experiments, each of which contained three replicates.

The current scenario shows paramount importance of PtNPs in human health and for the protection from various diseases caused by microorganisms. However, microorganisms are powerful and attain resistance to various antibiotics. Because of the recent increase in bacterial resistance, alternative therapeutic agents that are nontoxic to human beings but toxic to pathogenic microorganisms are urgently required. Therefore, the development of NP mediated antimicrobial agents is most warranted. Recently, several studies have focused on NP-based therapeutic agents against pathogenic bacteria. Metallic NPs such as, Pt, Ag, Pd, Cu, Au, ZnO, and TiO_2_ play a vital role in antibacterial activity against pathogens [160]. The antibacterial activity depends on NP morphology, size, and shape and also its surface charges. Most metallic NPs like Ag, Pt, Au, Pd, ZnO, and Cu have a negative zeta potential and thus have potential cell damaging properties. Although PtNPs have a more negative zeta potential and cause severe damage to the cells, they show enhanced antibacterial activity [161]. Apigenin functionalized PtNPs exhibited significant antibacterial activity against *Pseudomonas aeruginosa* and *Staphylococcus aureus* (Figure 15).

Previously, Rosenberg et al. [162] demonstrated that platinum electrolysis products inhibit growth of Gram-negative bacterium *Escherichia coli* and the multiplication of cell. Four years later, they again demonstrated that the square planar form of platinum was highly effective against rat sarcomas. Ma et al. [163] reported that a combination of quaternary ammonium-based antibacterial monomers with colloidal PtNPs potentially inhibits *Streptococcus mutans*. Polyaniline/Ag-Pt nanocomposite inhibits growth of *Staphylococcus aureus* [164]. Palladium complexes of polyamide S-containing sulfones showed the highest antibacterial activity against *Staphylococcus aureus* *and E. coli* and antifungal activity against *Aspergillums flavus* and *Candida albicans* [165]. Bimetallic NPs (AuPt) with sizes ranging between 2 and 3 nm have potent antibacterial activity against human pathogenic organisms such as *E. coli*, *Pseudomonas aeruginosa*, *Klebsiella pneumonia*, and *Salmonella choleraesuis* [166]. These reports suggested that bacterial growth inhibition is correlated with ATP production and mitochondrial membrane potential. Another recent report described that five different shapes and sizes of polyvinylpyrrolidone-coated PtNPs ranging between 2 and 20 nm were used to examine the antibactericidal activity against *P. aeruginosa*. They observed that NPs less than 3 nm in size were toxic to *P. aeruginosa* even at low concentrations, whereas NPs >3 nm in size showed no toxicity but only interacted with the bacterial membrane [167]. Two different sizes of PtNPs with PVP coating ranging between 5.8 and 57 nm were used to evaluate the antibacterial activity against *E. coli* and *S. aureus*. It was observed that the smaller NPs inhibited the proliferation of gram negative bacteria *E. coli*. These results agreed with those of a previous report [168]. Subsequently, Ahmed et al. [169] (2016) reported the antibacterial activity of PtNPs 2–5 nm in size against gram positive and gram negative bacteria. As a result, the PtNPs reduced bacterial cell viability through reactive oxygen species (ROS) production, led to membrane integrity loss, and also increased the survival rate of infected Zebra fish. Polyaniline/Pt-Pd nanocomposite shows potential antibacterial activity against *Streptococcus* and *Staphylococcus* *species*, *E. coli*, and *Klebsiella* spp. [170]. Platinum–PMMA nanocomposites (PtNCs) inhibit the cell viability of *Streptococcus mutans* and *Streptococcus sobrinus* [171]. Pt/Ag bimetallic NPs (BNPs) decorated on porous reduced graphene oxide (rGO) nanosheets exhibited increased antibacterial activity against *E. coli* on interfaces between metal compositions, rGO matrix, and bacteria. The release of nanocomposites led to a rapid release of silver ions, thus trapping the bacteria in the porous rGO matrix. Polyvinylpyrrolidone (PVP) as PVP/PtNP nanocomposites shows potential antibacterial activity against *E. coli*, *Lactococcus lactis*, and *Klebsiella pneumoniae* [172]. Biologically synthesized PtNPs using rind extract of the fruit of *Garcinia mangostana* showed antibacterial activity against *Staphylococcus* spp., *Klebsiella* spp., and *Pseudomonas* spp. (except *Bacillus* spp.). The highest activity was observed against *Klebsiella* spp. compared with other bacterial species.

## 7. Antifungal Activity of Platinum Nanoparticles

Commercial antifungal agents lead to side effects such as liver damage, nausea, renal failure, increase of body temperature, and diarrhea. At present, alternative therapy is required for recovery from fungal disease. Previously, Gardea-Torresdey et al. [81] reported silver NPs as having potential antifungal activity against spore-producing fungi. Recently, a study compared the antifungal activity of PtNPs and commercially available antifungal agents. The biofabricated PtNPs showed potential antifungal activity against different pathogenic fungi such as *C. acutatum*, *C.* *fulvum*, *P.* *drechsleri*, *D.* *bryoniae*, and *P.* *capsici* [94]. The persistence of the biopolymer mediated synthesized platinum nanocomposites (GKPtNPs) was assessed to analyze the antifungal activity against fungal strains such as *A. parasiticus* and *A. flavus*. They observed antifungal activity of the nanocomposite induced the morphology of the mycelia, membrane damage, increased the level of ROS, eventually leading to DNA damage and cellular death [173].

### 7.1. Anticancer Activity of Platinum

Cancer is a leading cause of death worldwide. According to the International Agency for Research on Cancer (IARC), the global cancer burden is estimated to have risen to 18.1 million new cases and 9.6 million deaths in 2018 (https://www.who.int/cancer). Every year, the number of cancer patients is increasing at an alarming level. It is projected that by 2030, approximately 13 million patients will be diagnosed with cancer and the estimated death rates are 13.1 million [174]. There are several conventional treatments used for the treatment of cancer such as chemotherapy, radiation, hormone therapy, and surgery. Although chemical treatment plays a major role in cancer therapy, chemotherapy is associated with some side effects. Radiation is end stage of therapy, and surgical therapy is associated with recurrence rate related to resection of tumors. Therefore, it is essential to develop alternative, simple, and effective therapy for prevention of cancer. NP mediated cancer therapy seems to be effective, simple, and free from undesired side effects.

Cisplatin (cis-[PtCl_2_(NH_3_)_2_], cis-diamminedichloridoplatinum(II)) and PtNPs have proven antitumor activity against various types of cancer cells. For instance, sarcoma 180 and leukemia L1210 cells were discovered by Barnett in the 1960 [175]; however, they involve dose-dependent toxicity and intrinsic or acquired drug resistance. According to Galanski et al (2005), platinum compound-based drugs have paved the way for cancer treatment, and 50% of cancer patients use platinum drugs for chemotherapeutic treatment [176]. A few decades ago, chemotherapeutic agents were used for treatment of several cancers. Most of these drugs are highly effective, but they also generate systematic toxicity and drug resistance to cancer cells [177]. Platinum derivatives like cisplatin, carboplatin, and oxaliplatin were used for treatment of cancer. All three drugs are square planar platinum (II) complexes surrounded by ligands: two amine ligands on the left side strong interaction with the platinum ion is called as non-leaving group and other two chloride ligands on the right side interaction with the platinum ion to form bonds with DNA bases [178]. Cisplatin is one of the major compounds used for the treatment of cancers such as small cell lung cancer, ovarian cancer, melanoma, lymphoma, myelomas, and colon and neck cancer [179]. It also used in the treatment of testicular cancer with a cure rate of above 95% [180]. Different sized NPs (<20, <100, and >100) have been used to analyze the toxicity in human colon carcinoma cell line (HT29). The results suggested that the toxicity effects depend upon the size of the PtNPs, but it is not due to the production of ROS [181]. Platinum ions were used as an anticancer therapeutic molecule with a similar strategy to cisplatin. Several reports suggested certain limitations of cisplatin such as nephrotoxicity [182], ototoxicity, neurotoxicity, hemolysis [183], and toxicity to gametogenesis [184]. Although chemist target platinum compound is considerably variation from structure activity relationship (SARs) based established nonclassical platinum compound such as poly platinum compounds, Pt (IV) prodrugs, complex with stereochemistry mechanisms, platinum-tethered intercalators, and monofunctional complexes, which is discerned from bifunctional cross link of classical compounds. The classical platinum anticancer agent, cisplatin, exerts its cytotoxic effect by selectively binding to the N7 atom in the purines of the DNA molecule and forms platinum-DNA adducts, which break the DNA double helical structure, impairing its replication and transcription [185,186]. These properties are exploited for the treatment of cancer and are used for the treatment of various types of cancers such as head, neck, brain, testicular, bladder [187], ovarian, or uterine cervical cancers [188]. However, platinum-based cancer therapy drugs generate some toxicity and side effects [189,190]. Previous studies have reported that PtNPs administrated to chicken embryos at 1–20 µg/mL did not affect the growth and development of the embryo. They also activated the apoptosis in brain cells as well as decreased the proliferation, suggesting the role of PtNPs in brain cancer therapy [191].

In the past two decades, nanomedicine has been growing immensely for detection of tumor and drug delivery system [192]. However, biosynthesized NPs play a very vital role in the molecular interaction and cross biological barrier without distressing normal cells. The biologically synthesized PtNPs from *Saccharomyces boulardii* showed anticancer effects against two different cell lines—squamous carcinoma cell line A431 and luminal breast cancer cell line MCF7 [193]. Similarly, Sahin et al. [100] reported that biosynthesized PtNPs exhibited efficient antitumor activity against the MCF7 cell line. They induced apoptosis through G0/G1 cell cycle arrest. Subsequently, Ghosh et al. [194] evaluated the synergistic effect of anticancer activity using combined metallic NPs. The anticancer activity of combined NP (PtNPs-PdNPs) showed a 74.25% refinement than that of individual metallic nanoparticle such as platinum (PtNPs-12%) and palladium (PdNPs-33%). In addition, early stage of apoptosis development was observed in HeLa cells. In vitro studies have demonstrated that PtNPs inhibited the growth of A549 cells in a dose-dependent manner, and the in vivo data showed that PtNPs at the mid and high doses effectively inhibited and delayed the growth of lung cancer in SCID mice [195]. PtNPs can potentially induce anticancer activity compared to chemotherapeutic drug, such as cisplatin by increasing the level of glutathione, superoxide dismutase activity, and malondialdehyde level, which is similar to treatment of hepatocellular carcinoma induced by diethylnitrosamine in rats [196]. Kutwin et al. [197] reported differential effects of PtNPs and cisplatin on viability of U87 glioblastoma multiforme (GBM) cells. The results showed that PtNPs have potential effects on cell viability and induce genotoxic and proapoptotic effects in U87 glioblastoma multiforme (GBM) cells comparable with cisplatin. Furthermore, NP-Pt decreased the weight and volume and induced pathomorphological changes of tumor tissue. Recently, Gurunathan et al. [198] demonstrated the anticancer properties of graphene oxide–green PtNPs (GO-PtNPs) on human prostate cancer cells (LNCaPs). GO-PtNPs increased the apoptosis and decreased the cell viability and proliferation by elevating ROS production. The increased ROS by GO-PtNPs caused membrane damage, mitochondrial dysfunction, and oxidative DNA damage.

### 7.2. Cytotoxicity of PtNPs in Cancer and Non-Cancer Cells

The unique physicochemical properties of NPs lead to their incorporation in many other applications [1]. Previously, several NPs were used in biomedical applications such as diagnostic assays [199], molecular imaging [200], implants [201], and drug delivery systems [202]. Recently, PtNPs have attracted great attention in the field biomedical applications such as nanomedicine [3], photothermal therapy [203], radiation dose enhancement [204], computed tomography (CT), and X-ray contrast [7]. The immense need of NP usage is accompanied by the corresponding increase in human exposure to these novel nanomaterials. In this area, nanotoxicology was founded to investigate the safety of nanomaterials and their usage in biological systems [205]. NP toxicity is determined by several aspects such as size, shape, surface area, catalytic activity, charges, and chemical composition of NP [206] and also depends on various shapes and method of entry into the cells.

**Figure 16:**
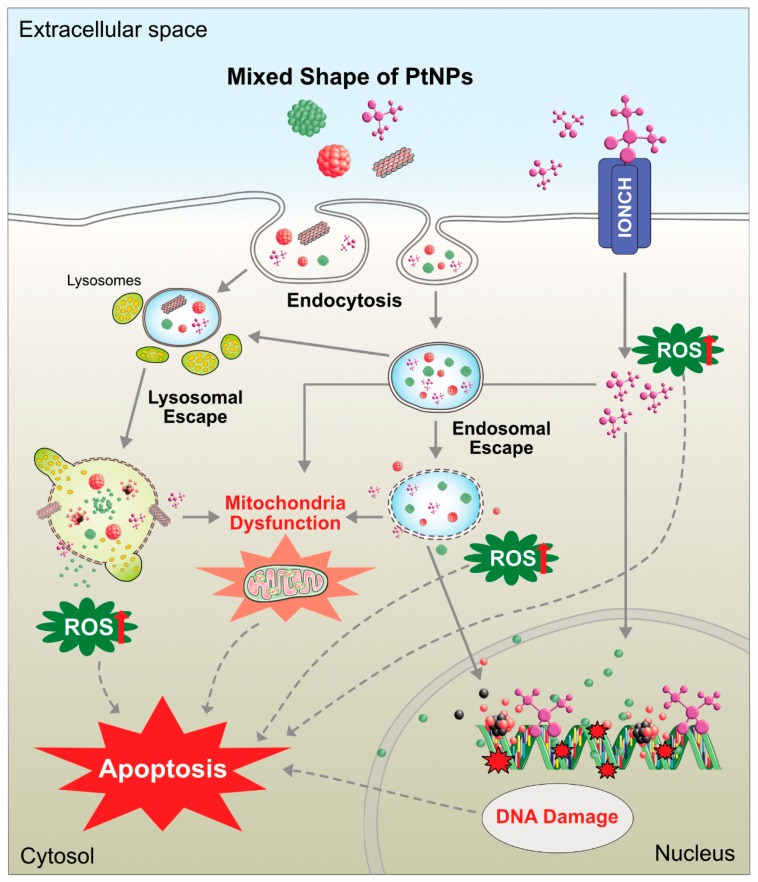
Diagrammatic sketch representing the possible mechanism of platinum nanoparticle induced cytotoxicity in cancer cell lines.

Commonly, toxicity analysis is carried out by two ways—in vivo and in vitro. These are all methods involved depends on system of toxicity. Previously, several studies documented size-based toxicity of NPs using various metallic NPs. Different metal NPs were employed for biomedical applications; particularly, gold and silver were used extensively for nanotoxicology investigation, but limited studies investigated the cytotoxicity of PtNPs. PtNPs were exposed two different types of cell lines, human endothelial cells and lung epithelial cells, for the toxicity analysis [207]. The results from this study indicated that both cells can uptake PtNPs, but there is no induction of ROS-induced cytotoxicity. The cytotoxicity of PtNPs is also depends on various shapes of the particles, the particle shape can determine the entry of particle and potentiation of cytotoxicity. The entry and effect of cisplatin and PtNPs are shown in Figure 16.

However, lung inflammation was observed in the respiratory tract. The cytotoxic nature of PtNPs was analyzed using tea capped PtNPs in cervical cancer cells (SiHa). The results showed that tea capped PtNPs influenced cell viability, nuclear morphology, and cell cycle distribution and inhibited proliferation of SiHa cells [208]. Onizawa et al. [209] synthesized polyacrylate stabilized PtNPs. These NPs were intranasally administered to DBA/2 mice exposed to cigarette smoke. The results revealed that PtNPs inhibited cigarette smoke induced depletion of antioxidant capacity, NF-κB activation, and neutrophilic inflammation in the lungs of mice. Gao et al. [210] analyzed cytotoxic properties of FePt@CoS(2) yolk–shell nanocrystals in HeLa cells; the results showed that FePt@CoS(2) yolk–shell nanocrystals displayed much lower IC(50) (35.5 ng/mL) than cisplatin (230 ng/mL). Toxicity against human breast cancer cells was reported using folic acid and poly(vinyl pyrrolidone) functionalized PtNPs using sodium borohydride in the presence of capping agents. The cytotoxicity was potentially higher than that of PVP-capped NPs [211]. Asharani et al. [212] reported 5–8 nm polyvinyl coated PtNPs that entered human cells by diffusion led to an increase in DNA damage, inhibition of proliferation of cells, and induction of activation of p21, leading to proliferating cell nuclear antigen-mediated growth arrest and apoptosis.

**Figure 17:**
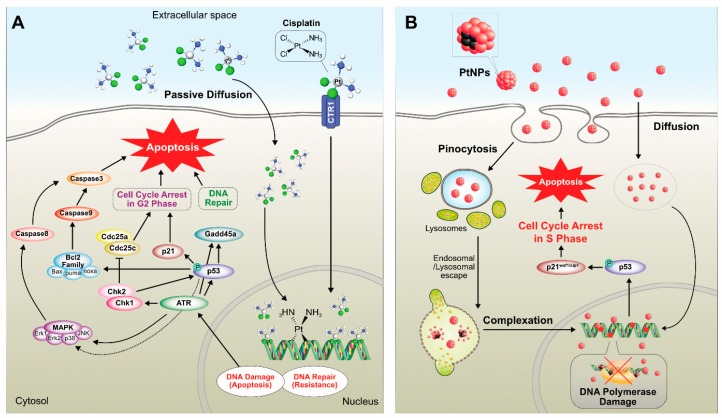
Schematic illustration showing the differential effect of cisplatin (**A**) and platinum nanoparticles (**B**) in cancer cells.

The effects of two different sizes of polyvinylpyrrolidone-coated coated PtNPs (5.8 nm and 57 nm) were analyzed on primary keratinocytes. Larger sizes of particles showed decreasing cell metabolism, but there was no significant effect on cell viability or migration, whereas smaller NPs exhibited more deleterious effects on DNA stability, and these triggered caspases [213]. Fibroblast cells L929 or macrophages RAW264 treated with PtNPs exhibited loss of cell viability and DNA damage, and PtNPs also inhibited matrix metalloprotease (MMP) activity [214]. Biologically synthesized PtNPs using pomegranate extract showed cytotoxic effects against MCF-7 cell line by decreasing cell viability and increasing apoptosis and DNA damage [100]. Two different types of PtNPs—NG-Pt NPs and MG-Pt NPS—with an average size of 15 nm and 8.5 nm, respectively, induced cytotoxicity in C2C12 cells by decreasing cell viability and increasing ROS generation. Furthermore, these PtNPs increased the expression of caspases 3 and 9 and also promoted the expression of proinflammatory proteins such as TNF-α, TGF-β, and NF-κB. Among these PtNPs, smaller-sized particles significantly increased cytotoxicity [215]. PtNPs induced apoptosis in Raw 264.7 cells by altering cell morphology and density and also increased apoptosis, activation of caspases 3 and 7, and DNA fragmentation [216]. Wei et al. [217] reported that polyethylene glycol-graphene quantum dots-Pt (GPt) with an average size of 5 nm sensitize oral squamous cell carcinoma (OSCC) in both normoxic and hypoxic conditions. GPt exhibits a strong inhibitory effect on tumor growth with less systemic drug toxicity and helps in combating hypoxia-induced chemoresistance in an OSCC xenograft mouse tumor model. Biologically synthesized PtNPs using leaf extract of *Azadirachta indica* altered cellular responses of HEK293 cells. PtNPs induce cytotoxicity of HEK293 cells in a dose- and time-dependent manner by increasing caspase 3 expression, depolarization of mitochondrial membrane potential, and DNA fragmentation [218]. Peptides stabilized with average diameters of 2.5 nm showed more potent toxicity against hepatic cancer cells (HepG2) than against other cancer cells and noncancerous liver cells compared to cisplatin [219]. A study reported the effect of novel platinum nanocomposite (PtNCP) beads on OSCC cell lines, and HSC-3-M3 cells were injected into nude mice both in vitro and in vivo. The results depicted that treatment with PtNCP beads suppressed tumor growth and identified increasing pathological necrotic areas. In vitro data suggest that PtNCP beads inhibited cell viability of HSC-3-M3 cells in a dose-dependent manner and induced cytotoxicity with increased leakage of lactate dehydrogenase [220]. Recently, Gurunathan et al. [101] reported that apigenin functionalized PtNPs exhibited potential cytotoxicity, genotoxicity, and proinflammatory responses in human monocytic cell line (THP-1) by increasing the levels of lactate dehydrogenase, generation of ROS, and production of malondialdehyde, nitric oxide, and carbonylated proteins and also increased apoptosis and oxidative DNA damage. Finally, PtNPs increased the expression of various proinflammatory cytokines such as interleukin-1β (IL-1β), IL-6, IL-8, tumor necrosis factor-α (TNF-α), granulocyte–macrophage colony-stimulating factor (GM-CSF), and monocyte chemoattractant protein 1 (MCP-1). All these findings substantially confirmed that PtNPs can induce cytotoxicity in both cancer and noncancer cells. Cisplatin and PtNPs are able to induce cytotoxicity differentially in various type of cancer cells (Figure 17).

## 8. In Vivo Toxicity of PtNPs

Asha Rani et al. synthesized different type of nanoparticles with various sizes (silver 3–10 nm, platinum 5–35 nm, and gold NPs 15–35 nm) using polyvinyl alcohol as capping agent and investigated the impact synthesized NPs. Polyvinyl alcohol capped silver NPs exhibited significant toxicity in Zebrafish embryos followed by PtNPs. No significant toxicity was observed with gold NPs. Silver and PtNPs revealed that AgNPs and PtNPs showed size-dependent toxicity in aquatic zebrafish embryos [221]. Recently, Claudia et al. [222] reported that citrate coated PtNPs increased the toxicity of mammalian liver cell line HepG2 at higher concentrations of PtNPs, whereas lower concentrations of PtNPs induced multiple stress response factors. PtNPs a caused size and dose-dependent effect on DNA strand breaks in human colon carcinoma cells (HT29) and increased the content of platinum in DNA [181,223]. Mice treated with PtNPs showed proinflammatory responses, and PtNPs increased various proinflammatory cytokines, such as IL-1, TNF-alpha, IL-6, IL-2, IL-12, IL-4, and IL-5, and concomitantly decreased intracellular levels of GSH [33]. Keratinocytes and mammary breast cells were incubated with folic acid functionalized PtNPs with an average size of 2–3 nm and showed significant toxicity toward cancer rather than non-cancer cells, suggesting that these PtNPs specially target cancer cells [224]. A comparative toxicity study between two different type of PtNPs such as sub-nanosized platinum particles (snPt) and nano-sized PtNPs was performed in the mouse liver. After intravenous administration of snPt into mice, the mice showed acute hepatic injury and increased levels of serum markers of liver injury and inflammatory cytokines. In contrast, administration of nano-sized platinum particles did not produce these abnormalities. Therefore, snPts have the potential to induce hepatotoxicity [225]. Further, authors demonstrated that single intravenous doses of snPt1 in in mice induce necrosis of tubular epithelial cells and urinary casts in the kidney, without causing any effect on lung, spleen, and heart, and cause a dose-dependent elevation of blood urea nitrogen, which is an indicator of kidney damage, whereas snPt induced significant cytotoxicity [225].

### 8.1. Use of PtNPs in Combination Therapy

PtNPs are potential drugs for combination therapy because of their salient features of accumulation of ROS and ROS scavenging properties in the treatment of complex diseases such as cancer and neurodegeneration. Combination therapy strategies have been used to promote synergetic efficacy and overcome the resistance of platinum drugs. For instance, the combination of platinum drugs and imaging agents allows the distribution of drug-loaded NPs inside the body and the tumor. Combination therapy has exhibited reduced systemic toxicity in comparison to either photothermal treatment or chemotherapy alone. Further, PtNPs can be exploited proper functionalization different reducing agents. Generally, platinum-based drugs are used in the treatment of cancer, including ovarian, head and neck, and lung cancer [226]. Platinum-based drugs yield positively charged, reactive aquated species that subsequently can form stable DNA-adducts and eventually cause cell death [226]. Nanocapsules of cisplatin enter the cells more efficiently than free compounds. Increased levels of platinum accumulation cause cisplatin-DNA-adduct formation in IGROV-1 cells [227]. A combination of PtNPs with irradiation by fast ions effectively enhances the strong lethal damage to DNA [204]. Nanocomposite S-containing platinum and titanium NPs exhibit very high photodynamic efficiency under a mild ultraviolet radiation in human cervical cancer cells. This combination potential seems to be more effective and a promising candidate in cancer treatment than TiO(2) and Au/TiO(2) NPs [228]. Bifunctional self-assembled NPs with a platinated fluorophore core with ultralow radiative transition synergistically induce photodynamic and photothermal therapy with tumor ablation through the generation of both singlet oxygen and the photothermal effect [229]. Multifunctional nano-platforms consist of iron-PtNPs (FePt NPs) with a polypyrrole (PPy) coating as a novel agent for combined photothermal therapy (PTT) and photoacoustic imaging (PAI). The obtained PPy-coated FePt NPs (FePt@PPy NPs) showed excellent biocompatibility and photothermal stability and high near-infrared (NIR) absorbance for the combination of PTT and PAI [230]. FePt-Cys NPs (FePt-Cys NPs) induced a burst of ROS, which suppressed the antioxidant protein expression and induced cell apoptosis through activation of the caspase system and impairment of DNA damage repair [231].

### 8.2. Biomedical Applications of PtNPs

**Figure 18:**
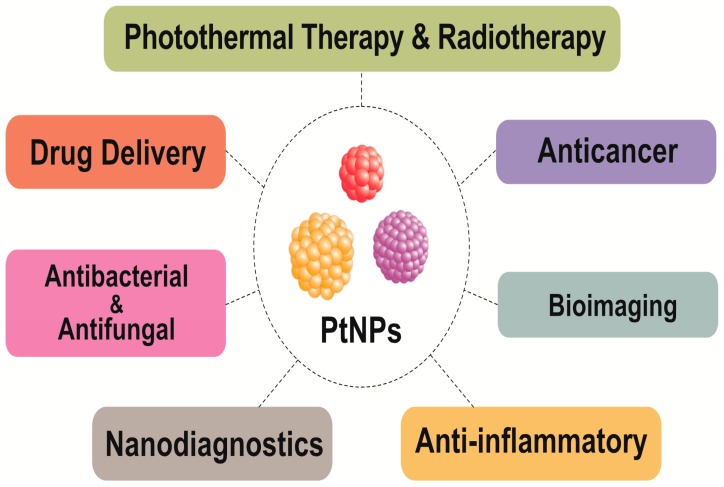
Multifarious applications of platinum nanoparticles.

PtNPs are used for various applications such as electro catalysts and catalytic converters, magnetic nanopowders, polymer membranes, cancer therapy drugs, coatings, plastics, nanofibers, and textiles (Figure 18). Herein, briefly, we discuss the biomedical applications of PtNPs. For instance, silica aerogel-supported PtNPs (SAP) were employed as fillers for preparing a self-humidifying Nafion-based composite membrane to enable the operation of PEMFCs without humidification subsystems. The synthesized SAPs were found to enhance the water adsorption and self-humidifying ability of the membrane because of their high surface areas and hydrophilic surfaces [232]. Four decades ago, thousands of new platinum drugs were synthesized and evaluated for their anticancer applications. However, only a small number of drugs were approved for marketing. In the past decades, more attention has been paid to the use of drug delivery and drug formulation methods of nanoplatin, lipoplatin, aeroplatin, and AP5346. The potential applications of nanocarriers for platinum drugs are prolonged drug circulation, cellular internalization of drugs, and multiple functional potential [233]. Platinum-based NPs act as a multidrug. They have a wide range of applications including cancer therapy, drug delivery, tumor targeting, co-encapsulation of therapeutic agents, and controlled release [233]. Drug delivery systems consist of two mechanisms—passive and active. Passive target systems focus on enhanced permeability and retention. The EPR system was described by Maeda and Mastumura [234,235], where they analyzed the accumulation of NPs in neoplastic tissues, which enhance the permeability and retention of NPs. The two essential characteristics of EPR, leaky vasculature and impaired lymphatic drainage, were characterized in solid tumor tissues [236,237]. Previously, several authors have reported that EPR effects increase the drug absorption as well as prolong drug retention [236,238,239]. Several nanocarriers have been developed for tumor-targeted drug delivery systems with different sizes and shapes of NPs.

However, the EPR effect enhances the tumor targeting of NPs. Several other parameters also influence the accumulation of NPs in tumors. Commonly, the put off blood circulation of NPs is prior to archive the successful drug delivery. Several polymers have been used as coating materials. PEG is frequently used as a coating material because of its size of <100 nm, high surface density, less accumulation in liver and spleen cells, reduced interaction with blood cells [240], and quick elimination of particles from circulation [241]. PEG has a prolonged blood circulation time, high tumor targeting capability, and improved tumor therapeutic efficiency [242]. Passive targeting is the accumulation of NPs in tumor cells, but the uptake of NPs by cancer cells through active targeting is facilitated through receptor or surface membrane proteins expressed on targets cells. A specific interaction between the ligands on surfaces of NPs and receptors expressed on the tumor-associated cells may facilitate the internalization of NPs through receptor-mediated endocytosis. Several targeting ligands have been used to actively target NPs including antibodies, antibody fragments, peptides, proteins, aptamers, and small molecules such as folic acid. Antibodies are used in nuclear medicine for diagnostic purposes and therapeutic applications. Antibodies are the target ligands for NPs because of their high selectivity, specificity, and specific cell type based on surface antigens [243]. Monoclonal antibody-mediated NP drug delivery systems involve human epidermal growth factor (HER2) 2, epidermal growth factor receptor (EGFR), transferring receptor (TfR), and prostate specific membrane antigen (PSMA). EGFR is a part of the ErbF family of receptors and is expressed in normal cells and over expressed in malignancy of epithelial cancer cells. Heparin-DDP (EHDDP) NPs drastically raise the intracellular concentration of platinum and Pt-DNA adducts in EGFR, over expressing non-small-cell lung cancer H292 cells. In vivo studies have also shown an increase in antitumor activity and improvement in pharmacokinetics and biodistribution with the conjugation of EGFR. Peptides are extensively used for targeting ligands based on their properties such as size, immunogenicity, stability, low cost, and conjugation properties. Different types of peptides have been used for specially binding to different targets on tumor cells such as small tripeptide RGD, which has a high affinity for Rvβ3 and Rv β5 integrins that are overexpressed in tumor cells [244]. Cyclic pentapeptide c enhanced the cytotoxicity of Pt (IV) NPs in PC3 and DU145 cells, but there was no enhancement observed in MCF7 cells. Folic acid is a small-molecular-weight molecule with more innate advantages such as scalability, reproducibility, easy conjugation process, low immunogenicity, low affinity, and a wide diversity [245]. Folate receptors (FR α and β) are also associated with membranes that are capable of transporting folate into the cell [246]. FR is upregulated in several human cancers such as ovarian, breast, endometrial, renal, lung, and colon cancers. Its expression is minimal in normal tissues [247,248]. As a result, the folate receptor is an attractive target ligand for anticancer agents [248]. Over 90% of FR-α is expressed in ovarian cancers [249], whereas FR-β is expressed in activated macrophages and malignant hematopoietic cells [250]. Previously, Dhar et al. [251] reported that folate Pt (IV)-SWNT conjugates showed higher cellular uptake in FR(+) JAR cells than that in FR(-) NTera-2 cells and observed higher cytotoxicity compared with that of cisplatin. Aptamers are short oligonucleotide ligands with different folding structures which can bind to specific biological targets [252]. The synthesized PSMA targeting aptamer functionalized Pt (IV) prodrug PLGA-PEG NP (Pt-NP-Apt) transport to cisplatin to prostate cancer cells. Pt-NP-Apt explained the in order to magnitude over effective than that of free cisplatin in PSMA+ LNCaP cells [253]. Cascade the Pt-NP-Apt showed equivalent antitumor efficacy in prostate cancer at 1/3 the dose of cisplatin and reduced accumulation of Pt in kidneys [254].

## 9. Conclusions and Future Perspectives

Platinum plays a crucial role in industrial applications such as a catalyst in fuel cells and in biosensors. Recently, Pt-based nanomaterials have attracted interest in both academic and industrial fields because of their unique features and function as nanocarriers, nanozymes, and nanosensors for diagnostic purposes. In this review, we discussed various methods for the synthesis of PtNPs including physical, chemical, and biological methods and provided a detailed account of biological methods. For the past decade, considerable progress has been made in the synthesis of monodispersed and well-defined structures of PtNPs with sizes ranging from 1.2 to several nm. Furthermore, we discussed the working principles and application of analytical techniques used for characterization of NPs. More importantly, we discussed the toxicological effect, biomedical applications, and use of PtNPs in combination therapy. For a long time, Pt-based materials have played a critical role in clinical research to overcome the undesired side effects of chemo- and radiation therapy. Thus, the diagnostic and medical industries are exploring the possibility of using PtNPs as a next-generation anticancer therapeutic agent. Although, biologically prepared nanomaterials exhibit high efficacy with low concentrations, several factors still need to be considered for clinical use of PtNPs such as the source of raw materials, the method of production, stability, solubility, biodistribution, controlled release, accumulation, cell-specific targeting, and toxicological issues to human beings. The development of PtNPs as an anticancer agent is one of the most valuable and warranted approaches for cancer treatment. The future of PtNPs in biomedical applications holds great promise, especially in the area of disease diagnosis, early detection, cellular and deep tissue imaging, drug/gene delivery, as well as multifunctional therapeutics. Furthermore, to overcome the obstacles in exclusive multidrug resistance, multifunctional PtNPs need to be designed for diagnosis and targeting and as nanocarrier and phototherapeutic agents. The current emphasis of molecular medicine is to develop more novel tools, which can be used for early-stage disease diagnosis and long-term availability in the cellular system. Integration of nanomaterials, especially PtNPs, could extend the construction of the theragnostic platform, which combines therapeutics with diagnostics, to make the diagnosis processes more simple and rapid and less invasive. Furthermore, progressive development of novel nanocomposites containing PtNPs with multifunctional modalities could lead to better ways to use PtNPs as nano-theragnostic entities in biomedicine.

As the usage of PtNPs shows immense potential in the medical field, various new modalities need to be developed. Although various methods are available to prove the efficacy of nanomaterials, the synergistic effects of PtNPs and low concentration of anticancer drugs on anticancer activity/tumor reduction are still obscure. Therefore, more studies are required to explain the synergistic effect of PtNPs with anticancer drugs at a single time point. These studies could provide an understanding of the mechanisms and efficiency of the synergistic effect of two different agents or multiple agents; thus, they would help to develop a novel system bearing multiple components with synergistic effects for the treatment of various types of cancer. Although PtNPs have been focused on for therapeutic purposes, further research is required in animal models along with multicenter studies to confirm the mechanisms and to gain a comprehensive picture of biocompatibility vs. toxicity of PtNPs. Finally, if we succeed in all these studies, it would help the researchers of the nanoscience and nanotechnology community to develop safer, biocompatible, efficient cancer or antiangiogenic agents containing PtNPs. In future, multifunctional PtNPs would be an attractive platform for biomedical applications and may change the business model of pharmaceutical industries.

## Appendix A

**Table A1:** Synthesis of PtNPs using various parts of biological templates and their sizes and shapes.

Name of the Organism	Parts Uses	Size (nm)	Shape	Ref.
**Bacteria**				
*Desulfovibrio vulgaris*	Periplasmic space/cellular surface			[71]
*Acinetobacter calcoaceticus*	Intracellular	2–3.5	Cuboidal	[72]
**Fungi**				
*F. oxysporum*	Extracellular	10–50	triangle, hexagons, square, rectangles	[74]
*Neurospora Crassa*	Intracellular	2–3, 4–35, 7–76 and 20–110	Quasi spherical, single crystalline and round nano aggregates	[80]
**Plants**				
*Doipyros kaki*	Leaf	2–12		[82]
*Fumariae herba*	Whole	30	Hexagonal and pentagonal	[83]
*Anacardium occidentale*	Leaf	100	Irregular rod	[84]
*Azadirachta indica*	Leaf	5–50	Small and large sphere	[85]
*Piper betle* L.	Leaf	2–0.4	Spherical	[86]
*Ocimun sanctum*	Leaf	23	Irregular	[87]
*Phoenix dactylifera*	Fruit	1.3–2.6	Spherical	[91]
*Lantana camara*	Leaf	35	Spherical	[93]
*Prunus x yedoensis*	Gum	10–20	Circular	[94]
*Camellia sinensis*	Leaf	30–60	Flower	[95]
*Antigonon leptopus*	Whole plant	5–190	Spherical	[96]
*B. prionitis*	Leaf	1–2	Monodispersed	[97]
*Punica granatum*	Peel	16–23	Spherical	[98]
	Pomegranate	20.12	Spherical	[100]
**Algae**				
*Padina gymnospora*		25	Octahedral	[172]
**Bacteria**				
*Saccharomy cesboulardii*	Intracellular	80–150		[193]
**Plants**				
*Eichhornia crassipes*	Leaf	3.74	Spherical	[255]
Quercus glauca	Leaf	5–15	Spherical	[256]
*Bacopa Monnieri*	Leaf	5–20	Spherical	[257]
*Cochlospermum gossypium*	Tree-Gum	2.4	Spherical	[258]
**Algae**				
*Plectonema boryanum* UTEX 485	Cell extract	<300	Spherical	[259]
**Bacteria**				
*Calothrixv* cyanobacteria	Intracellular and extracellilar	3.2		[260]
**Plants**				
*Pinus resinosa*	Bark	6–8	Irregular	[261]
*Gloriosa superb*	Tuber	0.83–3	Spherical	[262]
*Terminalia chebula*	Fruit	<4	Spherical	[263]
*Cacumen platycladi*	Whole plant	2.4-0.8	spherical	[264]
